# Project description and crowdfunding success: an exploratory study

**DOI:** 10.1007/s10796-016-9723-1

**Published:** 2016-12-07

**Authors:** Mi (Jamie) Zhou, Baozhou Lu, Weiguo (Patrick) Fan, G. Alan Wang

**Affiliations:** 1 Pamplin College of Business, Virginia Polytechnic Institute and State University, Blacksburg, VA 24061 USA; 2 School of Economics & Management, China University of Petroleum, Qingdao, 266580 China

**Keywords:** Crowdfunding, Content analysis, Persuasion, Empirical study, Predictive model

## Abstract

Existing research on antecedent of funding success mainly focuses on basic project properties such as funding goal, duration, and project category. In this study, we view the process by which project owners raise funds from backers as a persuasion process through project descriptions. Guided by the unimodel theory of persuasion, this study identifies three exemplary antecedents (length, readability, and tone) from the content of project descriptions and two antecedents (past experience and past expertise) from the trustworthy cue of project descriptions. We then investigate their impacts on funding success. Using data collected from Kickstarter, a popular crowdfunding platform, we find that these antecedents are significantly associated with funding success. Empirical results show that the proposed model that incorporated these antecedents can achieve an accuracy of 73 % (70 % in F-measure). The result represents an improvement of roughly 14 percentage points over the baseline model based on informed guessing and 4 percentage points improvement over the mainstream model based on basic project properties (or 44 % improvement of mainstream’s performance over informed guessing). The proposed model also has superior true positive and true negative rates. We also investigate the timeliness of project data and find that old project data is gradually becoming less relevant and losing predictive power to newly created projects. Overall, this study provides evidence that antecedents identified from project descriptions have incremental predictive power and can help project owners evaluate and improve the likelihood of funding success.

## Introduction

In recent years, crowdfunding has emerged as a revolutionary financing model that allows small entrepreneurs to raise funding in the early stages of their projects, particularly those that may otherwise struggle to obtain capital (Kuppuswamy and Bayus 2013; Belleflamme et al. 2014). Today, there are approximately 1250 active crowdfunding platforms across the world, which together channeled $16.2 billion in 2014, representing a 167 % increase from $6.1 billion in 2013 (Massolution 2015). Having their project successfully funded is crucial to project creators as it provides not only initial funds for project development but also access to valuable future resources, and eventually turn their projects into successful entrepreneurial organizations (Mollick 2014). Previous research shows that only 45 % of the projects on these platforms are successfully funded (Greenberg et al. 2013; Mollick 2014). As a result, identifying general antecedents of funding success (i.e., successfully funded) has been of great interest to researchers because it can provide insights to project creators to maximize their funding success (Greenberg et al. 2013; Xu et al. 2014).

It is natural to believe that one of important antecedents of funding success is the quality of project, and previous research on crowdfunding has suggested that project quantity is positively associated with the likelihood of funding success (Mollick 2014). However, the project quality is a latent construct and is measured from different aspects such as innovation, market condition, management team and so on. This measurement requires high level of expertise and experience in venture investment and it is usually done case by case. Consequently, existing studies on antecedent of funding success mainly focus on project properties that may directly or indirectly impact the funding success. For example, research has found that project properties^1^ such as the funding goal, campaign duration, number of Facebook friends of the project creator, etc. are associated with funding success (Agrawal et al. 2011; Greenberg et al. 2013; Z. Li and Duan 2014; Mollick 2014; Xu et al. 2014; Kuppuswamy and Bayus 2015).

Although existing research has identified an impressive list of antecedents that are associated with funding success, our primary criticism is the fact that they only focus on basic project properties, and that the information related to project descriptions is largely ignored. This paper tries to fill the gap by highlighting the importance of project descriptions and identifying influential antecedents of funding success under a theoretical guidance. Similar to traditional business plans, project descriptions are highly recommended to include the following information: what you are trying to do, how you will do it, how the funds will be used, qualifications to complete this project, people on the team, and how far along your project is (Kickstarter 2016). Previous entrepreneurship literature has evidenced that nascent entrepreneurs manage impressions and persuade business angels by manipulating language use of business plans (e.g., tone and style), in hoping to increase the likelihood of being selected for further consideration or getting funded (Chan and Park 2015; Parhankangas and Ehrlich 2014). Owners of crowdfunding projects are essentially entrepreneurs and have similar funding needs. We conjecture that they have the propensity to use project descriptions to promote their projects (products) and persuade backers to make a financial contribution.

Following the previous research on traditional business plans (Chen et al. 2009), we view the process by which project owners secure funding from backers as a “persuasion process” and introduce the unimodel of persuasion into crowdfunding domain. However, the primary interest of this paper is not to test unimodel of persuasion, but to utilize its theoretical guidance and explore potential antecedents of funding success. Although there are other persuasion theories such as Elaboration Likelihood Model (ELM), we choose unimodel because it clearly indicates the sources of influential factors and it has been successfully applied to entrepreneurship literature to study the persuasion process of venture capitalists’ funding decisions (Chen et al. 2009). Unimodel of persuasion classifies persuasive information into issue-relevant (the content of a message) and issue-irrelevant (cues other than the message itself), and it argues that these two types of information are functional equivalent in persuasion, though they may be quantitative different (Kruglanski 1989; Kruglanski et al. 2006; Chen et al. 2009). Guided by unimodel of persuasion, we identify five potential antecedents of funding success. Three of them (length, readability, and tone) are identified from the content of project description (issue-relevant) and two of them (past experience and past expertise) are from the trustworthy (issue-irrelevant) of project descriptions.

We then study whether these five newly identified antecedents are statistically influential on funding success and whether such influence is practically meaningful. Our logistic regression results show that each of these antecedents is significantly associated with funding success. When these five antecedents are incorporated into a predictive model (logistic), the results of N-Fold cross-validation tests indicate that the proposed model can predict funding success with an accuracy rate (F-measure) of 73 % (70 %). The average accuracy rate (F-measure) of the mainstream model is around 69 % (66 %), and baseline model around 59 % (57 %). This indicates that the proposed model has an improvement of roughly 14 percentage points (rounded) over the baseline model based on informed guessing and 4 percentage points improvement over the mainstream model based on basic project properties. The differences among these three models are statistically significant under the t-test. More importantly, considering that the mainstream model only beats the baseline model by 9 percentage points (57 % to 66 %), the 4 additional percentage points (66 % to 70 %) improved by our proposed model is fair significant, representing 44 % (i.e., 4 divided by 9) of mainstream’s performance over informed guessing. These results together show that our newly introduced variables have significant and practical impacts on the funding success of projects.

Additionally, crowdfunding environment has experienced tremendous changes since its inception, from perspectives such as platform functions, users and policy, and so on. For example, the numbers of users and projects have grown drastically (Kickstarter 2014b), which change the competition environment of crowdfunding. Additionally, both backers and owners are likely to change their behaviors through their use of the crowdfunding platforms. These changes make us wonder 1) whether the project data in earlier years have become “out of date” and have less power to predict funding success of future projects, and 2) whether the sub-sample of project data right before the projects being predicted contains the most relevant information and have higher predictive power. To answer these questions, we investigate the timeliness of project data and provide evidence that old project data is gradually becoming less relevant and losing predictive power to newly created projects. Overall, our results provide insights to researchers, project owners, and backers to better study and use crowdfunding platforms.

This rest of the paper is organized as follows. We first review literature related to the antecedents of crowdfunding success and the unimodel of persuasion. We then propose a new method based on the unimodel to quantify the influence of project descriptions based on content analysis. We present and discuss our empirical results using data sample collected from a popular crowdfunding site, Kickstarter. Finally, we provide conclusions and discuss opportunities for future research.

## Background and literature review

### Crowdfunding models and platforms

According to the context and nature of the funding effort, there are mainly four models of crowdfunding (Belleflamme et al. 2014). The first model is patronage-based, where supporters expect no direct return from their contributions or donations. The second one is lending-based, where the supporters expect some rate of return on their capital invested. The third one is reward-based, where supporters receive a reward for backing a project. The reward can simply be a mention/credit in a movie or a prototype (earlier version) of a product. The last one is equity based, where the supporters are treated as investors and are given certain shares of future profit of the project (Mollick 2014).

This study focuses on the reward-based crowdfunding, in which there are two dominant models regarding how funds are raised and distributed to project owners, represented respectively by two popular crowdfunding platforms, Kickstarter and IndieGoGo. Funds raised on Kickstarter follows a rule called all-or-nothing, which means no one will be charged for a pledge towards a project unless it reaches its funding goal (Kickstarter 2014a). On the other hand, IndieGoGo allows creators to keep the money pledged even the project fails to meet its goal (IndieGoGo 2014). While the all-or-nothing policy leads to greater motivation on Kickstarter, it’s nice to at least get some money as opposed to none. Kickstarter charge 5 % commission fee of the funds raised for each project and IndieGoGo between 4 and 9 % (when the goal is not met).

### Antecedents of funding success

Existing research has suggested that crowdfunding projects mostly succeed by narrow margins, or else fail by large amounts, and that crowdfunding success appears to be linked to project quality, i.e., projects of a higher quality level are more likely to be funded (Mollick 2014). However, the quality of a crowdfunding project is not easy to measure because individual backers generally lack relevant expertise owned by venture capitalists (VCs) and their contribution decisions are usually based on factors such as feeling and preference which, because of the limited backer data on the crowdfunding platform,^2^ are difficult to evaluate and quantify.

As an alternative, researchers turn to other factors that may directly or indirectly influence the funding success of a project. Some researchers find that project properties, such as project category, funding goal, and campaign duration, are associated with funding success. Others show that the existence of images or videos in project introduction is associated with funding success (Greenberg et al. 2013; Mollick 2014). Studies have also shown that a project owner’s social influence, proxied by the numbers of friends on social networks such as Facebook, has an impact on funding success (Mollick 2014). Furthermore, researchers find a strong geographic influence in crowdfunding projects: project owners are more likely to propose those projects reflecting the underlying cultural products of their geographic areas (e.g., a project related to country music in Nashville, Tennessee) (Agrawal et al. 2011; Mollick 2014). They suggest that the nature of the population in which founders operate is related to funding success (Kuppuswamy and Bayus 2013; Z. Li and Duan 2014). More recently, by studying the reciprocity effect on crowdfunding, Zvilichovsky et al. (2015) provide evidence that project owners’ backing-history has a significant effect on financing success: projects initiated by owners who have previously supported others have higher success rates, attract more backers and collect more funds.

### Project description and persuasion theory

Although existing research on antecedents and funding success contributes greatly to our understanding of crowdfunding, few studies have focused on the project descriptions. Research in venture literature has evidenced that “business plan serves as an important indicator of a venture’s potential for success.” (Chen et al. 2009, p. 202) Despite the difference between the funding environments, project descriptions are similar to traditional business plans in terms of both content and function (Kickstarter 2016). On the one hand, the project description is one of the most important information sources for backers to evaluate a project and make their funding decisions. Early-stage investments typically involve unproven technologies, unfinished products, and services. Thus, factual evidence pertaining to the new venture and its quality is often unavailable (Parhankangas and Ehrlich 2014). On crowdfunding platforms, backers “pre-order” products before their existence, and these products are “promised” to be delivered in a future day. Backers usually have no control over the project development, and there is little external information such as customer reviews for backers to evaluate a product or an owner. On the other hand, project description is one of the few available tools for project owners to communicate with potential backers and promote their projects. This is especially true before the project is launched. Given the fact that the number of crowdfunding projects is increasing dramatically in recent years, the competition for backers’ attention is becoming increasingly fierce (Mollick 2014). This highlights the importance of project descriptions for both project owners and backers on crowdfunding domain. We conjecture that project owners have the propensity to use project descriptions as marketing tools to influence potential backers’ contribution decisions.

There are only a few studies that have examined information content of project descriptions in the context of crowdfunding. These studies, however, either use a case study approach relying on small samples (Ordanini et al. 2011) or simply include all phrases as predictive variables (Mitra and Gilbert 2014). To identify the potential influential antecedents from project descriptions, we need to understand how information is processed by backers to form their funding decisions. On this point, social judgment and persuasion research offer potential insights. For example, Parhankangas and Ehrlich (2014) find the business angels’ funding decisions are influenced by the language use of business proposals (plans). In another study, Chen et al. (2009) use a persuasion theory of the unimodel and investigate the extent to which venture capitalists’ perceptions of “entrepreneurial passion” from business plans influence their investment decisions. Following their approaches, we conceptualize the process by which project owners secure funding from backers through project descriptions as a persuasion process, and employ the unimodel of persuasion to identify potential antecedents of funding success.

Although the unimodel differs from other established paradigms of persuasion such as dual-process model of ELM (Petty and Cacioppo 1986; Petty et al. 2002; Rucker and Petty 2006), it has received greater recognition and acceptance in the literature in recent years (Chen et al. 2009; Catellani and Alberici 2012; Suárez-Vázquez and Quevedo 2015). Dual-process models suggest that influence is formed from two routes, namely, central route and peripheral route, and that the influence of two routes is both qualitatively and quantitatively different. In other words, individuals have a higher motivation or cognitive ability tend to rely more on the central route, and the influence of central route is more enduring than that of the peripheral route. Unimodel of persuasion also classifies information into two types: issue-relevant (the content of a message) and issue-irrelevant (cues other than the message itself) (Chen et al. 2009). However, unimodel only suggests the quantitative difference, not the qualitative difference, of the influence of different information. In other words, unimodel assumes that the processing of issue-relevant information and issue-irrelevant information share the same route (individuals subjectively decides which information qualifies as their basis for persuasion-based decisions), and they have the same enduring effect on individual’s decision.

Consistent with previous research on the influence of business plans on venture capitalists’ funding decisions (Chen et al. 2009), we believe that the unimodel explains better the backers’ decision-making process because it emphasizes the subjectivity and equality of information basis and parsimoniously captures the persuasion process. In the context of crowdfunding, a backer’s funding decision is determined by what the backer believes to be the basis for his/her judgment. For example, if a backer personally knows the project owner, he/she may rely less on the project description itself and use his/her personal experience as the basis to make a funding decision; otherwise, the backer may be more likely to use project description as the basis to make the decision. In addition, both persuasion and funding decision on crowdfunding are not a “one-time” thing. Backers usually get to know the project at a different time; Kickstarter provides backers with tools (web pages) to monitor projects they have backed (Kickstarter 2014a), and backers can re-visit the project descriptions and get “influenced” throughout the campaign. More importantly, Kickstarter allows backers to make and change their decisions (contribute, cancel, or re-contribute) anytime before the campaign is ended (Kickstarter 2014a). In other words, project description is accessed by backers at the different time point (or multiple times by a backer), and their funding decision can be formed at any time point before the campaign is ended. However, the ending time of a campaign is fixed and is the same to every backer; this setting makes the enduring effect of influence less meaningful in the context of crowdfunding. In summary, unimodel of persuasion provide us with a theoretical guidance regarding the information sources of potential antecedents of funding decisions, without dealing the subtle details of influencing process.

In this study, the potential antecedents of funding success are investigated at the aggregated level (backer population), not at the individual backer level. Unimodel of persuasion is an individual level theory and mainly links the influential antecedents to individual backer’s funding decision. Following the previous literature (Baum et al. 2001; Baron 2008; Chen et al. 2009), we extend this link to include funding success at the aggregated level. Unimodel suggests that a antecedent can have a strong influence on a backer’s funding decision. We argue that, however, if a antecedent has an influence on enough backers’ funding decisions,^3^ at the aggregated level, these funding decisions will lead to a higher likelihood of funding success. Previous persuasion and venture literature have evidenced a link between entrepreneur’s traits and venture success and growth (Baum et al. 2001; Baron 2008; Chen et al. 2009). Especially, using the unimodel of persuasion, Chen et al. (2009) find that the affective and cognitive passion revealed from traditional business plans are positively associated with venture success. They further explain that these traits can make “entrepreneurs more persuasive,” thus “these entrepreneurs had a higher probability of achieving success in new ventures.” (Chen et al. 2009, p. 201) The argument we used to extend the link is also consistent with marketing and advertising literature that persuasion (or response) occurs at individual level, but the overall success (of marketing and advertising) is evaluated at the aggregated level (e.g., market-level sales) (Sun et al. 2010; Venkatraman et al. 2014).

## Methodology

Existing research on crowdfunding success are generally interested in evaluating the performance (predicting accuracy) of different models, assuming each model using the same set of antecedents. For example, Greenberg et al. (2013) evaluates the performance of various decision tree algorithms and support vector machines with different kernel functions. Specifically, they evaluate the performance of radial basis, polynomial and sigmoid kernel functions with varying costs for support vector machines. For decision trees, they further evaluate different learning algorithm variations such as J48 Trees, Logistic Model Trees, Random Forests, Random Trees and REPTree. Then they choose the highest performing set of algorithms and boost them using the AdaBoost algorithm to see if accuracy is improved. In other words, existing research focuses on the model level, trying to find the best models with optimized parameters to achieve the highest performance. Our study, on the other hand, focuses on the antecedent level and tries to identify additional antecedents from a theoretical perspective (i.e., the unimodel of persuasion), and evaluates whether they have the incremental power to predict funding success. These additional contributing antecedents can then be applied to different predictive models.

### Identifying exemplary antecedents of funding success

This study introduces the unimodel of persuasion into crowdfunding domain. However, the primary purpose of this study is not to test the unimodel theory, but to use the theory as a guidance and identify potential antecedents of funding success beyond basic project properties. In addition, this study doesn’t mean to identify a complete set of antecedents from project description, rather, we use exemplary antecedents to demonstrate that unimodel can be used to facilitate our understanding of persuasion process and uncover potential antecedents. We choose exemplary antecedents based on following criteria: 1) the antecedent must be closely related crowdfunding context; 2) the antecedent must be aligned with unimodel of persuasion; 3) the antecedent can be reliably extracted or calculated automatically, and 4) the antecedent must be widely used in literature.

Unimodel of persuasion suggests that backers’ funding decisions are influenced by the content of project description (issue-relevant message) and cues other than project description itself (issue-irrelevant message) (Chen et al. 2009). Since the project description of crowdfunding project shares the similar content and role of traditional business plans, we identify potential antecedents based on previous research on traditional business plans (or investment proposals). For the content of project description, research on traditional investment proposals finds that language use can positively influence business angels’ decision and increase the likelihood of being funded (Parhankangas and Ehrlich 2014). So we first identify three exemplary antecedents based on language use of project description through a content analysis. Similarly, for the cues other than project description itself, research on traditional business plans find that entrepreneur’s traits such as tenacity, proactivity and passion are associated with venture success and growth (Baum et al. 2001; Chen et al. 2009). So we identify two exemplary antecedents based on project owners’ traits. These exemplary antecedents are discussed in more detail below.

The three exemplary antecedents identified from the language use of project description are length, readability, and tone. Length captures the amount of information project owner provided. Since crowdfunding projects typically involve unproven technologies, unfinished products, and services, there is litter external information regarding the factual evidence pertaining to their final products and quality. Project owners thus are encouraged to provide sufficient information for backers to evaluate the project, increase backers’ confidence, and earn their trust (Kickstarter 2016). So we posit that length is a potential antecedent of funding success. Readability (not legibility) captures the easy of understanding of project description. Because of its importance on effective communication, readability has been advocated by the government, business, and other organizations. For example, The U.S. Department of Defense uses the Reading Ease test as the standard test of readability for its documents and forms, and Florida requires that the readability of life insurance policies must be greater than a set score (Wikipedia 2015). So we posit that readability is also a potential antecedent of funding success. Lastly, tone captures the general attitude used by project owner to describe their products or services. Research on business plans has evidence that entrepreneurs use moderate positive language in order to attract investors (Parhankangas and Ehrlich 2014). So we believe that tone could also be a potential antecedent of funding success.

The two potential antecedents identified from project owners’ traits are past expertise and past experience. These two traits demonstrate the competence and trustworthy of project owner and increase their likelihood of funding success. Because of potential costs, backers very often do not want to support a project that is unlikely to succeed (Li and Duan 2014). These costs can be associated with backer’s disutility from having his or her money locked in the project or potential risk that rewards cannot be delivered as promised. Past expertise captures the achievement and success of the previous project creating actives. Higher expertise generally leads to a higher likelihood of funding success. Past experience captures project owners’ previous backing and creating activities on the crowdfunding platform. On the one hand, backing a project will make a project owner think from a backer’s perspective, thus having a better understanding of information needs of backers. This experience may increase his/her competence to create a better project description. On the other hand, research has evidenced a reciprocity effect on crowdfunding that project owner with more backing activates are more likely to receive reciprocal backing from project owners they backed (Zvilichovsky et al. 2015). In other words, project owners can accumulate social capital by backing projects and at least partially cash it out when they raise funds for their own projects. In either case, past backing activities are likely to increase funding success. Additionally, project owners can learn from both successful and failed project they created in the past by enhancing the strengths and improving weaknesses. As a result, past creating activities are also likely to impact funding success. In summary, both past expertise and past experience are potential antecedents of funding success.

### Predictive model and measures

Because each project can be classified as either a success (reaching the funding goal when the campaign is completed) or a failure (not reaching the funding goal when the campaign is completed), we build a logistic regression model to study the influence of project descriptions on the success of a funding project. We use logistic regression model, instead of other binary classification models, for the following reasons. First, as discussed above, our primary purpose is to identify additional contributing factors, not to evaluate the model performance. So the selection of model is based on whether the model has the capability to evaluate factors. Second, we want to analyze each newly introduced factors quantitatively and see whether they have significant and incremental predictive power to funding success. The significance test of coefficients of logistic regression model provides us with such a capability. Third, logistic regression model has widely used in binary classification and has competitive performance results with other traditional classification models such as Support Vector Machines (Hua et al. 2007; Abrahams et al. 2014). All variables or antecedents used in this study are organized and described in Table 1.

**Table 1:** Variable (antecedent) description

Type	Source	Variable	Definition
Newly Identified Antecedents	Content of project description	length	The number of word contained in project description.
		readability	The ease of understanding of project description, measured by using readability index.
		tone	The ratio of positive and negative words in project description.
	Trustworthy cues (project owner traits)	pastExperience	The number of projects previously created and backed by the owner
		pastExpertise	The rate of projects successfully funded.
Previously Identified Antecedents	Control Variables	goal	Project goal, the amount owner seeks to raise using crowdfunding.
		duration	The number of days for which a project accepts funds.
		FBConnected	Whether the project owner has linked or created a Facebook page for the project.
		FBFriends	The number of Facebook friends a project owner has.
		numImages	The number of images embedded in the project page.
		numVideos	The number of videos embedded in the project page.
		rewards	The number of pledge levels
		year	The year a project launched ,not the year the project completed.
		category	The category of the projects.

As shown in Table 1, the potential antecedents that influence funding success are arranged into two categories, namely, previously identified and newly introduced antecedents. Those newly introduced antecedents are further organized into content and trustworthy cue of project descriptions, according to unimodel of persuasion. In order to evaluate the incremental contribution of those newly introduced antecedents in determining funding success, we control for other major antecedents of funding success identified in previous research, such as project category, goal and duration (Greenberg et al. 2013; Mollick 2014). Our model is shown below.

1 $$ \begin{array}{l} logit(Success)={\beta}_0+{\beta}_1 length+{\beta}_2 readability+{\beta}_3 tone+{\beta}_4 ton{e}^2\\ {}+{\beta}_5 pastExperience+{\beta}_6 pastExpertise+{\beta}_7 goal\\ {}+{\beta}_8 duration+{\beta}_9 rewards+{\beta}_{10} numImages\\ {}+{\beta}_{11} numVideos+\varepsilon \end{array} $$

Where *success* takes a value of either 0 or 1, indicating whether a project is successfully funded. Following the previous literature, we measure the amount of information (*length*) by using the number of words in the project description (Wang et al. 2011; Zhou et al. 2015). We measure the ease of understanding (*readability*) by calculating the readability score of the project description. Specifically, we use Gunning fog index (hereafter Fog Index) to measure the readability of project description. The Fog index was developed by Robert Gunning (“Gunning fog index,” 2015) and has been widely used in literature (F. Li 2008; Wang et al. 2011; Zhou et al. 2015). Fog index proposes that, assuming everything else to be equal, more syllables per word or more words per sentence make a document harder to read. For example, texts such as formal financial reports generally have a Fog index greater than 16 (F. Li 2008), and documents for a wide audience generally need a fog index less than 12 (“Gunning fog index,” 2015). Please note that a higher value of the Fog index corresponds to a lower level of readability. So we intentionally reverse the sign of Fog Index to reflect the direction. Specifically, readability is calculated as follows:

2 $$ readability=-\left[\left(\frac{words}{sentences}\right)+100\times \left(\frac{complex\  words}{words}\right)\right]\times 0.4 $$

Following the tone management literature (F. Li 2008; Davis et al. 2012; Huang et al. 2013), we measure the *tone* as the percentage difference of positive and negative words in the project description. Specifically, it is calculated as follows:

3 $$ tone=\frac{positive\  words- negative\  words}{total\  words} $$

The positive and negative words used in the formula are defined in Harvard-IV dictionary, which has been widely used to measure the tone reflected in textual contents (Davis et al. 2012; Huang et al. 2013). Because of the nature of marketing and persuasion, we expect that the project description usually has a net positive tone. In other words, more positive words than negative words are used in project descriptions. However, previous research has found that the venture capitalists preferred “moderate use of positive language” and evidenced a curvilinear relationship between positive language and funding success. So we add a quadratic term of tone to the model.

As the reason discussed above, since project owners can learn and benefit from both their backing and creating activities, we measure past experience (*pastExperience*) by using the number of projects previously either created and backed by the owner, before the one being investigated. Finally, we measure past expertise (*pastExpertise*) by using the ratio of total funds raised and total goals required for all previous projects ended before the one being investigated. For example, assuming an owner has created three projects; when we investigate his past expertise by the time he is creating the third project, we should only consider the total funds raised and the total funds required for the first two projects, assuming both projects have been completed.

## Empirical results

### Data sample

We collected real crowdfunding project data from Kickstarter.com to carry out our empirical analysis. We use Kickstarter mainly for two reasons. First, Kickstarter is a popular and prevalent crowdfunding platform. Founded in 2009, Kickstarter has become one of the largest crowdfunding platforms in the world. It has more than nine million backers, and three million of them are repeat backers. As of today, more than 93,000 projects have been successfully funded, and more than two billion dollars have been raised (Kickstarter 2015). Second, the majority of research on crowdfunding uses Kickstarter data (Greenberg et al. 2013; Li and Duan 2014). This makes the comparison of our results against those previous reported more meaningful and reliable.

Kickstarter doesn’t provide a pubic API (Application Program Interface), and the non-live projects (e.g., completed, canceled, etc.) are not directly searchable. However, live projects are organized in category and are convenient to navigate. Our data collection mainly consists of two steps. First, started in late 2012, we scraped the those “live” projects from Kickstarter websites by using a specially developed crawler. The crawler visited the website every other day and captured all live projects newly launched. Second, we scraped project data in early years based on those live projects already collected. Project’s profile page contains the historical projects created and backed by the owner, and comments and updates contain backers’ information which leads to other projects they backed. Similar to the approach used by Zvilichovsky et al. (2015), we used those “live” projects as seed and recursively iterate from projects to backers and backers to projects until the number of newly discovered projects per iteration converged. This step was only performed once a while when a big enough number of new projects were scrapped.

Our data sample covers all the projects from 2009 to November 2014. We excluded those funding projects that were still ongoing (6559 projects). In addition, we excluded those projects that were canceled (15,116 projects), purged (36 projects), and suspended (584 projects). Purged and suspended projects were usually handled by Kickstarter according to its policy or terms of use. Projects were canceled by owners for a variety of reasons. It’s possible that majority of projects were canceled because they were unlikely to reach the funding goals and project owners want to avoid the dismal end (stonemaiergames.com 2013). A brief examination also finds that many projects were canceled because they were simply testing projects, with unreasonable low funding goal (e.g., $1, $2) or duration (e.g., 1 day). And some projects were canceled because project owners want to make necessary improvement and re-launch the project, or “amazing partners reached out” during the campaign (needwant.com 2016). It’s also interesting to find out some projects were canceled even after successfully funded because of unforeseen changes from either project owners or backers (themarysue.com 2016). These projects were not treated as failed projects because they were not typically failed projects. We also followed a previous study (Mollick 2014) and removed those projects with a funding goal below $100 (1982 projects) or above $1,000,000 (294 projects), because those extremely small or large projects may have characteristics very different from the majority of projects. We finally removed those projects with less than 100 words in their descriptions, because, upon inspection, they are either incomplete or represent non-serious efforts to raise funds. Our final data sample consists of 151,752 projects across all 15 funding categories. These steps are summarized in Table 2.

**Table 2:** Sample selection and description

Sample projects	
All projects scraped	183648
Less live projects	6559
Less canceled, purged and suspended projects	15736
Less projects with goal less than 100 or above 1,000,000	2276
Less projects with invalid project description (missing, less than 50 words, etc.)	7325
Total sample projects	151752

Table 3 shows the descriptive statistics for variables used in this paper. On average, the projects in our sample have an average funding goal of $15,126, with half of them less than $5000. The average (median) campaign duration is 34 (30) days; 47 % of projects have at least an image, with the average (median) number of 4.67 (1); and 80 % of projects have at least a demo video, with the average (median) of 1.18 (1). The results also show that the descriptions have an average (median) length of 646 (482) words, are generally positive (with a net positive tone), and are easy to understand (with fog index around 13). In addition, although owners usually have some past experience, with several projects backed or created, their past expertise is very limited. Averagely, they have raised 22 present of required funds on their previous projects, if any (more than 75 % of projects are created by the first-time owners).

**Table 3:** Descriptive statistics

Variables	Min	1st Q	Median	Mean	3rd Q	Max
length	100	296	482	646.00	812	30280
readability	-19.68	-14.69	-12.92	-13.08	-11.33	-5.10
tone	-0.22	0.04	0.07	0.07	0.10	0.48
pastExperience	0	1	3	6.00	6	1267
pastExpertise	0.00	0.00	0.00	0.22	0.00	9.97
goal	100	2000	5000	15126	12000	100000000
duration	2	30	30	34.00	39	90
rewards	1	6	9	9.00	12	250
FBFriends	0	24	383	503.00	755	7797
hasImage	0.00	0.00	0.00	0.47	1.00	1.00
hasVideo	0.00	1.00	1.00	0.80	1.00	1.00
numImages	0	0	1	4.67	6	248
numVideos	0	1	1	1.18	1	25

In the follow sections, we present our empirical analysis results from three aspects. First, we briefly discuss the current status of crowdfunding on Kickstarter from different angles such as category and year. Second, we evaluate the increment influence of newly identified antecedents and report the practical improvement when they are included to predict funding success. Third, we investigate the timeliness of project data and provide evidence that old project data is gradually becoming less relevant and losing predictive power to newly created projects.

### Descriptive results

#### Overall funding status by category

Tables 4 and 5 present the status of crowdfunding by category on Kickstarter. Overall, the success rate of our sample projects is 46 %, which is comparable to what reported by Kickstarter (Kickstarter 2015). Most of the basic project properties vary across 15 categories. Table 4 shows that in term of project number, the top three categories created are Film & Video, Music, and Publishing; and the least three are Dance, Craft, and Journalism. However, a project attractive to owners doesn’t necessarily mean it is also attractive to backers. For example, Dance is one of the three categories that are least attractive to owners but has the highest success rate among all the categories, possibly because of low fund required and less market saturation (competition). On average, a project requires raising a fund of $14,541. Technology and Games require the highest funds, $39,073 and $27,520, respectively; Music and Crafts require the least funds, $7289 and $5794, respectively. It is also reasonable to notice that, generally, project categories requiring higher (lower) funds have lower (higher) success rates.

**Table 4:** Previously identified antecedents by category*

Category	Project	Success	Goal	Success rate	Funding ratio	Backers	Duration
Art	12,255	6130	9160	50 %	0.84	45.82	33.10
Comics	4274	2307	8466	54 %	1.24	147.32	35.79
Crafts	1873	633	5794	34 %	0.96	33.85	30.93
Dance	2064	1452	7302	70 %	0.89	48.19	32.71
Design	8500	3585	23,385	42 %	1.76	280.39	34.21
Fashion	6781	2135	11,986	31 %	0.73	76.97	32.68
Film & video	34,194	15,334	20,417	45 %	0.62	71.28	35.77
Food	7781	2832	19,493	36 %	0.67	77.85	33.76
Games	10,023	4235	27,520	42 %	1.85	417.15	33.39
Journalism	1101	395	11,833	36 %	0.53	58.91	35.14
Music	28,662	16,989	7289	59 %	0.81	61.90	35.71
Photography	4846	1767	8179	36 %	0.59	46.55	34.72
Publishing	17,117	5942	9037	35 %	0.61	55.14	34.28
Technology	6235	1997	39,073	32 %	1.28	262.84	34.83
Theater	6046	4051	9178	67 %	0.85	53.19	33.83
Overall	151752	69784	14,541	46 %	0.95	115.82	34.06

*Because of space limitation, not all previously identified antecedents are listed

**Table 5:** Newly identified antecedents by category

Category	Length	Readability	Tone	Past experience	Past expertise
Art	551	-13.53	8.41	6.08	0.19
Comics	719	-12.32	6.81	15.16	0.37
Crafts	527	-12.41	8.35	6.62	0.28
Dance	500	-14.94	10.68	5.44	0.18
Design	844	-12.67	7.33	9.68	0.34
Fashion	595	-12.53	8.20	5.23	0.25
Film & video	637	-13.43	5.89	5.52	0.10
Food	651	-13.03	8.44	5.47	0.09
Games	1124	-12.46	8.43	18.46	0.99
Journalism	563	-14.00	7.31	5.07	0.05
Music	453	-12.59	7.81	4.65	0.18
Photography	533	-13.81	7.13	5.41	0.09
Publishing	679	-12.96	7.01	6.47	0.11
Technology	985	-13.42	7.04	6.97	0.20
Theater	503	-14.22	6.68	4.81	0.14
Overall	657.67	-13.22	7.70	7.40	0.24

Mollick (2014) reports that crowdfunding projects mostly succeed by narrow margins but fail by large amounts. However, we found this is more noticeable in popular categories with a significant number of projects. For example, Untabulated results show that only 3 % of the funded projects in Film & Video category have a funding ratio greater than 2 (two times the required fund) while that percentage is around 25 % in Comics and design category. Duration has little variation among different categories, with a range from 33 and 36 days, possibly because that Kickstarter has a default duration of 30 days and limits it up to 60 days (it was 90 days before June 2011). Surprisingly, there are some projects have a duration less than 5 days. Further investigation finds they are mostly small project, with funding goals less than $500.

Table 5 presents the data related to new antecedents derived from project descriptions. Projects in Games has the longest description (11,24 words) and those in Music has the shortest (453 words). It is also worth noting that the overall readability is high (with Fog index around 13). Possible reason is that comparing to traditional business plans, project descriptions are more likely to be written the informal language. Both past experience and past expertise vary greatly across categories, reflecting the different popularity and competition among categories.

#### Overall funding status by year

Tables 6 and 7 present the status of crowdfunding by year on Kickstarter. We find the project properties are relatively more stable over time than across categories. The results show a clear trend that the number of projects and backers and the amount of goal are all increasing over time, though the speed is decreasing. This is reasonable because, as more users join Kickstarter and become more familiar with the platform, more projects are created. In addition, the mutual trust between project owners and backers increases with the familiarity and maturity of Kickstarter platform, thus, more expensive ones are likely to be funded in later years. The results also shows that the durations before 2011 are higher, which is consistent with the fact that Kickstarter allows a duration up to 90 days before June 2011 but reduces to 60 days afterward.

**Table 6:** Previously identified antecedents by year*

Category	Project	Success	Goal	Success rate	Funding ratio	Backers	Duration
2009	1145	551	5846	48 %	0.70	36.55	58.66
2010	9314	4486	6857	48 %	0.69	42.53	46.75
2011	22686	11607	9242	51 %	0.84	59.49	40.19
2012	36485	17133	15,066	47 %	0.86	112.89	33.41
2013	40975	19777	17,206	48 %	1.02	145.51	32.02
2014	41147	16230	18,483	39 %	0.85	111.40	31.85
Overall	151752	69784	12,117	46 %	0.82	84.73	40.48

*Because of space limitation, not all previously identified antecedents are listed

**Table 7:** Newly identified antecedents by year

Category	Lengh	Readability	Tone	Past Experience	Past Expertise
2009	405	-13.03	7.14	3.65	0.03
2010	399	-13.19	6.69	4.37	0.04
2011	432	-13.01	7.00	6.44	0.11
2012	593	-12.90	7.38	6.62	0.14
2013	806	-13.17	7.30	8.47	0.29
2014	718	-13.18	7.60	8.15	0.32
Overall	558.72	-13.08	7.18	6.28	0.15

The results in Table 7 show that, although readability and objectivity of project descriptions are relatively stable, project owners are disclosing more information through project description over time, with an exception in 2014. Specifically, the length of project descriptions increases from 405 to 718 words. Another interesting finding is that increasing trend of both past experience and past experiment. As shown in the results, the value of past experience increase consistently from 3.65 in 2009 to 8.15 in 2014, and the value of past expertise also increases consistently from 0.03 in 2009 to 0.32 in 2014. This is reasonable because that, over time, project owners gain experience and expertise by backing and creating more projects, and by making projects more persuasive thus more likely to raise funds.

### Impact of project descriptions on predicting funding success

#### Logistic regression results

In order to investigate whether those newly identified antecedents have incremental influence on funding success, we run two logistic models. Model A represents the mainstream model which only includes antecedents identified by previous research from basic project properties (i.e., control variables); Model B represents the proposed model which also includes those exemplary antecedents identified by this study. The results are reported in Table 8 below.

**Table 8:** Logistic testing results of antecedents

	Model A (Mainstream)	Model B (Proposed)
Antecedents(IVs)	Coefficient (t-statistics)	Coefficient (t-statistics)
Intercept	2.75*** (32.51)	-0.27*** (−2.64)
log(length)		0.38*** (31.84)
readability		-0.05*** (−19.75)
tone		2.72*** (8.99)
tone^2^		-17.85*** (−9.93)
log(pastExperience)		0.68*** (74.3)
log(pastExpertise)		0.58*** (20.75)
log(goal)	-0.6*** (−113.18)	-0.64*** (−110.02)
duration	-0.01*** (−30.69)	-0.01*** (−26.25)
log(rewards)	1.24*** (83.12)	0.97*** (61.64)
log(FBFriends)	0.04*** (21.79)	0.01*** (5.85)
log(numImages)	0.28*** (41.18)	0.11*** (14.21)
log(numVideos)	0.83*** (42.18)	0.7*** (34.23)
year	controlled	controlled
category	controlled	controlled
Pseudo R-square (%)	17.16	22.98

**, *** statistically significant at 5 % and 1 %, respectively

As shown in Model B (proposed model), consistent with unimodel of persuasion, we find antecedents identified from both content of project descriptions (*length, readability, and objectivity*) and owner traits of project descriptions (*pastExperience and pastExpertise*) are significantly associated with funding success. Specifically, we find for every 1 % increase of length, the log odds of funding success increases by 0.38; this number is 0.68 and 0.58, for 1 % of increase of past experience and expertise, respectively. We find tone is positively associated with funding success. However, the quadratic term of tone has a negative coefficient, which indicates the curvilinear relationship between tone and funding success. In other words, moderate use of positive tone can demonstrate project owners’ confidence and optimism, thus, increases the likelihood of success. Excessive use of positive tone, however, may weaken project’s credibility and lead to an adverse effect. The results are consistent with those reported by Parhankangas and Ehrlich (2014). Finally, we find readability is negatively associated with funding success, with 1 % of its increase reduce the log odds by 0.05. This is puzzling because we expect a positive association since a more readable project description is easier to be understood by potential backers. However, as discussed in the descriptive statistics section, project descriptions are mainly written in informal language with low Fog index (easy to understand). Under this circumstances, formally written project descriptions may signal the preparedness and professionalism of project owners (Chen et al. 2009), thus increase the positive perception of backers and increase the likelihood of funding success. Similar results have also been reported by a previous study (Luo et al. 2013).

The results of other antecedents are consistent between Model A and B. For example, a higher requirement of funding goal and a longer campaign duration are negatively associated with funding success; a higher number of reward levels has a positive influence on funding success; and as expected, a higher number of Facebook friends, images, and videos are also positively associated with funding success.

#### Evaluation of predicting performance

We first compare the predicting performance of our proposed model (Model B) with the mainstream model (Model A) discussed above. We use the entire data sample to train (via logistic models) and test the predicting performance. In addition to the accuracy rate, we also use F-measure to evaluate the prediction performance. F-measure considers both prediction accuracy and recall accuracy and thus provides a balanced performance evaluation (Ferri et al. 2009; Sokolova and Lapalme 2009; Powers 2011). The results are reported in Table 9.

**Table 9:** Performance measures and confusion matrix

Panel A	Model A (Mainstream Model)			
Accuracy:	69.34 %		F-Measure:	66.20 %
		Predicted		
		Failed	Success	Total
Actual	Failed	TN = 59666 (72.79 %)	FP = 22302 (27.21 %)	81968
	Success	FN = 24226 (34.72 %)	TP = 45558 (65.28 %)	69784
	Total	83892	67860	151752
Panel B	Model B (Proposed Model)			
Accuracy:	73.09 %		F-Measure:	70.31 %
		Predicted		
		Failed	Success	Total
Actual	Failed	TN = 62,556 (76.32 %)	FP = 19412 (23.68 %)	81968
	Success	FN = 21426 (30.7 %)	TP = 48358 (69.3 %)	69784
	Total	83982	67770	151752

As shown in Table 9 Panel B, the proposed model has a predicting accuracy (F-measure) of 73.09 % (70.31 %), while the mainstream model has a predicting accuracy (F-measure) of 69.34 % (66.20 %). This indicates the proposed model has a better performance of predicting funding success. In addition, the confusion matrixes of both models further demonstrate that proposed model has higher true positive and true negate rates (69.3 % and 76.32 %) than those rates (65.28 % and 72.79) of the mainstream model. Correspondingly, the proposed model has a lower false positive and false negative rates (23.68 % and 30.7 %) than those rates (27.21 % and 34.72) of the mainstream model. These results show that the newly identified antecedents have incremental predictive power.

However, training and testing a predictive model using the same data sample is not a good practice. A recommended practice is to use different datasets to train and test the model (Bengio and Grandvalet 2004). In this step, besides the proposed and mainstream model, we include another model called baseline model. Baseline model is based on informed guessing. In baseline model, we classify each project as “success” or “failure” simply according to the overall probability of funding success. For example, if 40 % projects are successfully funded, the overall probability of funding success is 40 %. Therefore, each project will be classified as “success” with a probability of 40 % and to “failure” with a probability of 60 %. Then we calculate the prediction performance by comparing projects’ assigned status values (i.e., success or failure) with their true status values.

For each predictive model, we employ N-fold cross-validation test (with N set as 3, 5 and 10) to evaluate the prediction performance. The N-fold cross-validation test has been widely used to validate the performance of classification (Bengio and Grandvalet 2004; Li 2008). For each N, our data sample is randomly divided into N parts, then N experiments are performed, with N-1 parts used as training data for the predictive model to classify the remaining part. The average prediction performance is reported for the given N. Our results of N-fold cross-validation test are reported in Table 10.

**Table 10:** N-Fold Cross-validation tests accuracy (F-Measure)

N	Baseline (%)	Mainstream (%)	Proposed (%)
3	59.34 (57.26)	69.21 (66.58)	73.36 (70.59)
5	59.67 (57.19)	69.36 (66.15)	73.28 (71.32)
10	59.83 (57.68)	69.67 (66.49)	73.19 (70.43)

The results show that our proposed model achieves the highest performance, with the average accuracy rate (F-measure) around 73 % (70 %). The average accuracy rate (F-measure) of the mainstream model is around 69 % (66 %), and baseline model around 59 % (57 %). This indicates that the proposed model has an improvement of roughly 14 percentage points over the baseline model based on informed guessing and 4 percentage points improvement over the mainstream model based on basic project properties. The differences among these three models are statistically significant under the t-test. More importantly, considering that the mainstream model only beats the baseline model by 9 percentage points (66 % vs. 57 %), the 4 additional percentage points (70 % vs. 66 %) improved by our proposed model is fair significant, representing 44 % (i.e., 4 divided by 9) of mainstream’s performance over informed guessing. These results together show that our newly introduced variables have significant and practical impacts on the funding success of projects.

**Fig. 1:**
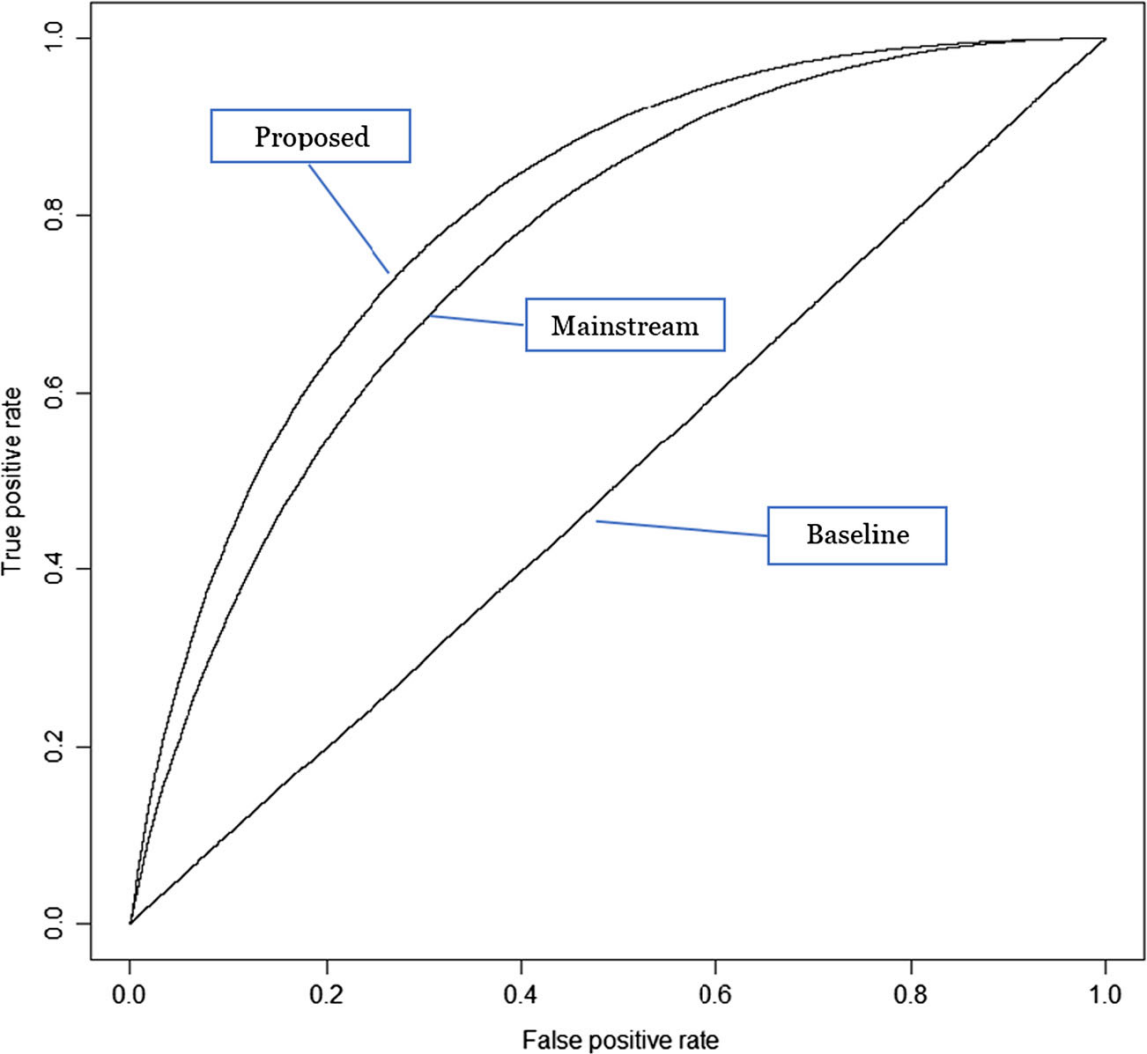
The ROC curves of baseline, mainstream, and proposed models

Both accuracy and F-measure are designed for the overall performance of predictive models. Sometimes, however, we need more specific information to make the funding decision. This is especially true when we evaluate prediction performance from the perspective of project owners. Because of the limited time and resources, project owners may not be interested in the overall success rate. Instead, they care more about whether their projects, if predicted as success, will truly be successfully funded. In another word, they want a predictive model that has high true positive rate and low false positive rate. Although this has been reported in the confusion matrixes, one of the visual illustration is to use ROC (Receiver Operating Characteristic) curve, which plots the true positive rate against the false positive rate at various threshold settings (Fawcett 2006). The results are illustrated in Fig. 1.

As shown in Fig. 1, comparing to the mainstream model, our proposed model has an ROC curve more convex toward upper-left. This indicates that the proposed model has a higher true positive rate and a lower false positive rate, which is more useful for project owners to change their project settings and evaluate the likelihood of funding success before projects are launched.

### Predictive power of project data over time

In the previous subsections, in order to ensure the comparability, we follow the previous studies and conduct our analysis by ignoring the temporal (i.e., time) information of projects. In other words, all projects are put in a single pool and predictions are in both directions: older project data are used to predict newer projects and, at the same time, newer projects are used to predict older projects. This can be clearly seen from our N-fold cross-validation test, in which the total sample is randomly divided into N parts without considering the creating time of each project. When we predict a funding success of a project, however, the only project data available to make a prediction is those historical data before the one being predicted, and it seems unreasonable to use future project data to train the predictive model and predict the funding success of past projects.

On the other hand, as a new platform of crowdfunding, Kickstarter has experienced great changes since its inception, from perspectives such as system functions, platform policy, and so on. In addition, both backers and owners have changed their behaviors greatly though their use of the crowdfunding platform. Furthermore, the number of users and projects grow drastically over time, which changes the competition environment of crowdfunding. These changes make us wonder whether the project data in earlier years have become “out of date” and have less predictive power to future project success, and whether the sub-sample of project data right before the projects being predicted contains the most relevant information and have higher predictive power.

In order to answer these questions, we slice the whole sample (2009 to 2014) by month and construct narrower, but relatively big enough subsample (e.g., 6 or 12 month^4^) and analyze the effectiveness of project data of different subsamples on prediction performance. We construct six subsamples consisting of one year’s project data from 2009 to June, 2014 (the last sub-sample contains only 6 month’s data), each of these sub-samples is used as training data to predict the funding success of projects between July and November of 2014 (our data sample ends in November 2014).

**Fig. 2:**
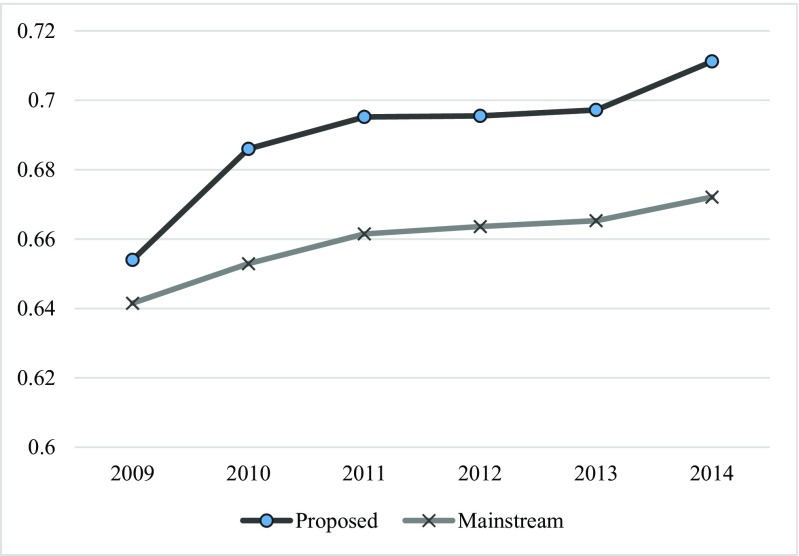
The timeliness of project data on predicting performance (F-Measure)

Figure 2 presents the results of the prediction performance (F-measure) by using each year’s data from 2009 to 2014. Consistent with our conjectures, we find that, overall, the prediction performance increases over time for both mainstream and proposed models. We remove the informed guessing model from this analysis because its performance only depends on the success rate of each year, which increases and then decrease as evidenced in Table 6. The figure indicates there are two bigger jumps in the year 2010 and 2014. In addition to the reason that project data in 2009 is the oldest relative to projects in 2014, another possible reasons is that, since Kickstarter was funded in 2009, the project data in 2009 may contains more noises and inconsistency. These two reasons together may result in the prediction performance by using data of 2009 is “much” lower than other years. The performance jump in 2014, on the other hand, may mainly reflect the timeliness of data because the first half year’s project data is used as training data to predict the projects of the second half year. The results also show that, from 2010 to 2013, although the improvement is relatively small, the overall trend of prediction performance is obviously increasing. These results together provide evidence that the historical project data is gradually becoming less relevant and losing predictive power to newly created projects. We also replace the F-measure with the accuracy rate and find the similar pattern.

## Conclusions and discussions

The success of crowdfunding warrants its importance of research, and we expect an increasing use of crowdfunding in future venture investment. By using a large dataset obtained from Kickstarter, a popular crowdfunding platform, we examine the influence of project descriptions on funding success. To do so, we rely on the unimodel of persuasion and identify five exemplary antecedents from project descriptions: three of them are related to the content of project descriptions and two of them are related to the owner traits of project descriptions. We then investigate the influence of these antecedents by using logistic model. Our results show that the proposed model can predict funding success with an accuracy rate of 73 % (or 70 % in F-measure), which represents an improvement of roughly 14 percentage points over the baseline model based on informed guessing and 4 percentage points improvement over the mainstream model based on basic project properties (or 44 % improvement of mainstream’s performance over informed guessing). These results together show that those antecedents identified from project descriptions have significant and practical impacts on the funding success of projects. We also investigate the timeliness of project data and provide evidence that old project data is gradually becoming less relevant and losing predictive power to newly created projects.

This paper contributes to the crowdfunding literature in several ways. First, to the best of our knowledge, this study is among the first to explore crowdfunding with a focus on the information content of project descriptions. Second, this paper is also among the first to introduce communication theory in general and persuasion theory in particular (i.e., unimodel) into the crowdfunding context. Using content analysis, we measure the influence of project descriptions and investigate its impacts on funding success. The newly identified antecedents from project descriptions can then be used in different predictive models to enhance the predicting performance. Third, the results reported in this paper highlight the importance of project descriptions and provide insights for project owners to understand the influence of antecedents and increase their funding success with proper balance among antecedents. Fourth, existing predictive models of funding success usually employ overall accuracy to measure performance (Etter et al. 2013; Greenberg et al. 2013; Mitra and Gilbert 2014). Our proposed model is also evaluated using more balanced performance measures (i.e., F-measure and ROC curve) to better serve backers to make funding decisions. Taken together, our results provide meaningful insights to researchers, project owners, and backers to better understand the importance of project descriptions and their influence on funding success.

This study is subject to several limitations. First, we conduct our studies merely base on one crowdfunding platform. There are other popular platforms (e.g., IndieGoGo) with different rules. This limits the generalizability of our results. Second, we mainly consider the information communicated between owners and backers through the crowdfunding platform (within-platform activities). There are many channels of “offline” communication and interactions between owners and backers (off-platform activities) such as media coverage, which are also critical to funding success. Third, we limit our study to the information content of project description before project launch. The information content of project updates and comments after project launch are also important to funding success. Fourth, we simply exclude cancelled projects from our study, which may cause our results biased.

This study also provides valuable opportunities for future research. First, some of the limitations can be addressed in future research. Future research can compare different crowdfunding platforms and gain more insights. Kickstarter and IndieGoGo have different rules regarding how to keep funds raised. These difference may lead to different motivations when selecting platform and different strategies when preparing the campaign. This study can also be extended to the stage after the project is launched and provide real-time monitoring and suggestions to increase funding success for project owners. Second, future studies can examine the influence of project descriptions by using more advanced features such as linguistic structures or methodologies such as topic modeling. Third, our study is conducted at a broader level to identify antecedents beyond basic project properties. Future study can further focus on a single antecedent and provide us with more thorough understanding. For example, Zvilichovsky et al. (2015) focus on past experience and find there exist a reciprocity effect on crowdfunding. Similar studies can be conducted based on other antecedents such as tone and readability. Fourth, with the fast growing of crowdfunding projects, it is becoming increasingly difficult for a project owner to attract enough backers and for a backer to choose a suitable project. Future research can work on bringing the two types of participants closer by identifying potential backers to owners or recommending potential projects to backers. Fifth, the main purpose of this study is to highlight the important influence of project descriptions and identify exemplary antecedents. In order to further improve the prediction performance, future research can incorporate those new antecedents into other classification models such as decision trees and Support Vector Machine (SVM), which provide better model calibration. Sixth, cancelled projects are excluded from study, which may make our results biased. Cancelled projects count for around 8 % of our data sample, they are mainly “perceived as failed” by project owners. Kickstarter allows project owners to cancel a project during and even after the campaign is ended. However, we know little regarding the influence of cancellation on project owners, backers and the general crowdfunding practice. Last but not least, the theory and methodology used in this study can be extended and applied to equity-based crowdfunding domain. We encourage future research to explore these areas and advance our knowledge in crowdfunding.

## Footnotes

1 In this study, the terms property, antecedent, factor, and variable are interchangeable, unless otherwise specified.

2 For example, Kickstarter only provide the total number of bakers, the information of each backer is not provided.

3 Theoretical, decisions can be both positive and negative. In this study, we mainly focus on positive funding decisions, in line with the role of project descriptions.

4 The length (number of months) can be changed accordingly, if necessary.
